# Pulmonary Valve Endocarditis With Tetralogy of Fallot: A Comprehensive Exploration

**DOI:** 10.7759/cureus.58013

**Published:** 2024-04-11

**Authors:** Anugya A Taksande, Krupa Bhanushali, Amar Taksande, SreeHarsha Damam, Amol Lohakare

**Affiliations:** 1 Department of Pediatrics, Jawaharlal Nehru Medical College, Datta Meghe Institute of Higher Education & Research (Deemed to be University), Wardha, IND

**Keywords:** cyanotic chd, infant, pulmonary valve, infective endocarditis, tetralogy of fallot

## Abstract

Infective endocarditis, a fatal infection with rising morbidity and mortality rates among infants and children, is characterized by microbial infection within the endocardium, the inner lining of the heart including heart valves. The heightened susceptibility to infection in children is attributed to pre-existing pathologies, structural defects, and comorbidities. This report details a case of a one-year-old child with tetralogy of Fallot, showcasing isolated pulmonary valve vegetations as a distinctive manifestation of infective endocarditis.

## Introduction

Infective endocarditis (IE) is an infrequent occurrence, particularly in the pediatric age group, with an incidence of approximately 0.43-0.69 cases per 100,000 children per year [1]. IE includes acute and subacute bacterial endocarditis as well as nonbacterial endocarditis caused by fungi and other microbiologic agents. Viridian-type streptococci and *Staphylococcus aureus* are the most commonly found bacteria responsible for infection in the pediatric age group.

Right-sided endocarditis is more common in patients admitted to intensive care units having peripheral and central catheters in situ or pacemaker-implanted patients or patients with certain skin or gynecological infections. In rare cases, it can also occur in patients with congenital cardiac defects with left to right shunt having bacteremia [2]. Factors such as injection drug use (IDU) and healthcare-associated risks have emerged as predisposing factors for congenital heart disease (CHD) patients to develop IE. Cyanotic CHD is characterized by intracardiac right-to-left shunting, leading to systemic circulation of unsaturated blood, resulting in arterial hypoxemia, pulmonary vasoconstriction, polycythemia, coagulopathy, and an increased risk of IE, along with the potential for brain abscess due to a heightened risk of paradoxical embolism. The risk of IE in cyanotic CHD is more than six times that in acyanotic CHD [3,4]. Clinical suspicion and echocardiography are pivotal in diagnosing IE in patients with predisposing factors. Here, we describe a case of tetralogy of Fallot (TOF) with pulmonary valve vegetation in an infant.

## Case presentation

A one-year-old female child presented with complaints of fever, cough, and cold persisting for one week. Medical history included TOF. Born to a primiparous mother via normal vaginal delivery, the infant who was 2 kg at birth, was identified with a heart defect during the mother's seven-month antenatal scan. After delivery, the mother observed poor feeding and poor weight gain and the infant was diagnosed with TOF at the age of one month through two-dimensional echocardiography.

In the current presentation, the child was admitted due to a week-long history of fever, cough, and cold. On admission, the patient had a temperature of 38.5°C, pulse rate of 118 beats/minute, blood pressure of 86/56 mmHg, respiratory rate of 30 breaths per minute, and oxygen saturation of 84% at room air in all four limbs. Physical examination revealed central cyanosis, grade II systolic murmur in the left second intercostal space, and a single-second heart sound. Laboratory investigations were done (Table 1).

**Table 1:** Laboratory Tests

Parameters	Results	Reference range
Hemoglobin	9.3 g%	11-14 g%
Total leucocyte count	8500/ mm^3^	5000-15000/mm^3^
Platelet count	1.18 lacs/ mm^3^	2-4.9 lacs/mm^3^
C reactive protein	39.87 mg/L	0-5mg/L
Serum sodium	138 mmol/L	137-145 mmol/L
Serum potassium	3.5 mmol/L	3.5-5.1 mmol/L
Serum urea	14 mg/dl	15-36 mg/dl
Serum creatinine	0.7 mg/dl	0.52-1.04 mg/dl

**Figure 1:**
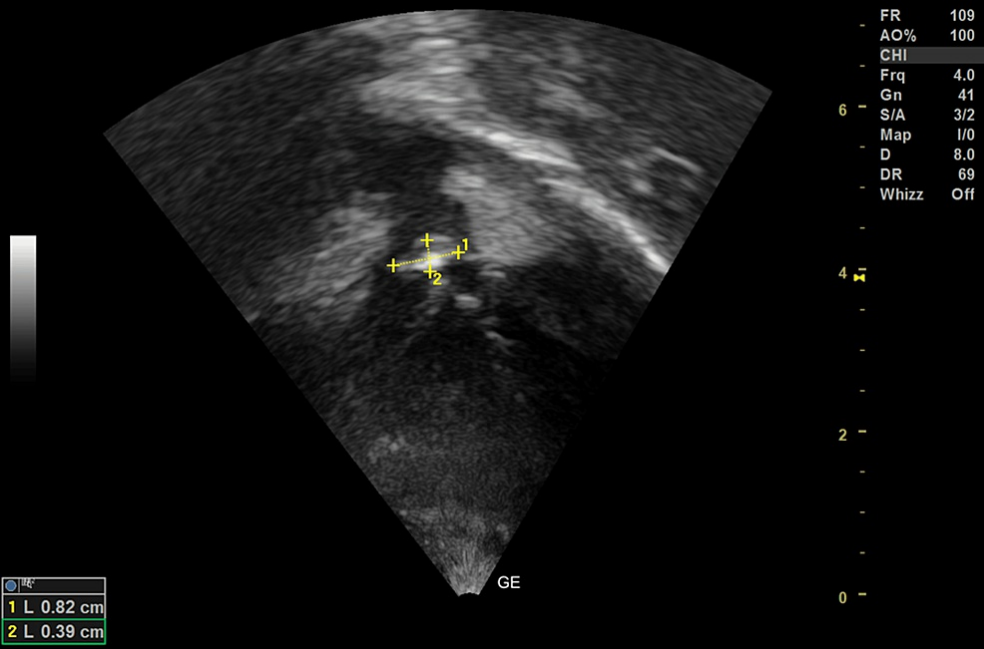
Subcostal view shows an 8x4 mm vegetation over the pulmonary valve

The patient's urinalysis showed no hematuria. Her coagulation profile was normal. The chest X-ray showed decreased pulmonary plethora with normal heart size (with cardiothoracic ratio 0.5). On echocardiography, the subcostal view revealed an 8x4 mm vegetation over the pulmonary valve (Figure 1).

**Figure 2:**
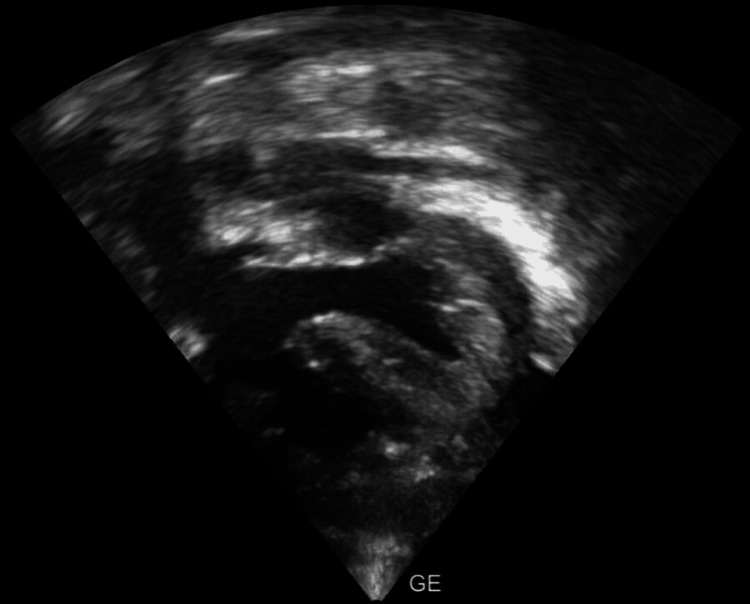
Subcostal long axis view shows large ventricular septal defect with overriding of the aorta

The subcostal long-axis view showed a large ventricular septal defect with overriding of the aorta (Figure 2).

Three blood cultures from right brachial vein, left brachial vein, and left femoral vein were sent over a period of one week, reporting the presence of *S. aureus *in the second and third culture reports. Blood culture results, echocardiography findings, and the clinical presentation indicated a diagnosis of IE. The child was initiated on injection ceftriaxone at 100 mg/kg/day in two divided doses for four weeks. The child responded positively, with no fever spikes noted after the sixth day of admission. In addition to antimicrobial therapy, propanolol was initiated to manage hemodynamic instability, aiming to optimise cardiac function. Iron supplementation was also provided as part of the supportive care. As the IE was effectively managed with antimicrobial therapy, a plan for surgical repair of tetralogy of Fallot with pulmonary valve repair/replacement was established. The child was discharged with advice for follow-up after 15 days, along with an explanation of warning symptoms.

## Discussion

The colonization and proliferation of microorganisms over the mural or valvular endocardium result in IE, which, on the right side of the heart, has an incidence of about 11%. In this situation, there is tricuspid valvular IE that is sometimes accompanied by endocarditis of the pulmonary valve. The presence of congenitally abnormal pulmonary valves predisposes children to endocarditis involving the pulmonary valve. Intravenous drug abuse is the most common causative factor for the same in adults [5,6].

TOF is a cyanotic congenital heart disease characterized by four distinct features: ventricular septal defect, right ventricular hypertrophy, pulmonary stenosis, and overriding of the aorta [7]. Children with TOF are more prone to developing IE, a condition marked by infection of the heart's inner lining, primarily affecting the valves. Isolated cases have been observed in individuals undergoing hemodialysis and orthotopic liver transplants [8,9]. The structural abnormalities inherent in TOF, including pulmonary stenosis and overriding of the aorta, may contribute to alterations in blood flow dynamics, creating an environment conducive to infective endocarditis. While right-sided endocarditis is less common than left-sided, TOF patients can exhibit unique susceptibility due to the complexity of their congenital heart defects. The rarity of right-sided endocarditis often results in missed diagnoses, and cohort studies suggest causative organisms include *S. aureus*, *Streptococcus viridans*, enterococci, and coagulase-negative staphylococci, along with gram-negative bacilli [4,5,8]. The clinical presentation of IE varies, and it should be suspected in cases of sepsis of unknown origin or fever in the presence of risk factors. It can manifest as fever alone or directly present with complications such as systemic emboli or stroke. Following the American Heart Association (AHA) guidelines, both transthoracic echocardiography (TTE) and transesophageal echocardiography (TEE) are essential in the initial evaluation and subsequent follow-up of many IE patients [10]. TTE's estimated sensitivity and specificity are 30-63% and 91-100%, respectively, while TEE's are 87-100% and 91-100%, respectively.

Robbins et al. found that vegetation size could predict the response to medication alone [11]. Vegetations < 10 mm had a 100% response rate to medication, compared to 63% in vegetations > 10 mm, leading to unavoidable surgery for the remaining patients. The assumption was that as bacterial colonies deepen, their metabolism and proliferation slow down, making certain antibiotics less effective. Our patient had a definitive diagnosis of IE according to the modified Duke criteria, with two major and two minor criteria [12]. In a retrospective study, Di Filippo et al. found that the most frequent targets for IE include unrepaired complex cyanotic CHD, CHD which is corrected using prosthetic material, and small ventricular septal defects [13]. The management of IE primarily involves prompt diagnosis through blood culture and echocardiography, followed by medical treatment with antibiotics based on sensitivity. Early initiation of antibiotic therapy based on blood culture sensitivity is paramount. The choice and duration of antibiotics should be individualized, considering the microbiological profile. Surgery is considered in select cases to prevent embolism, address valve dysfunction that may lead to heart failure, and rarely in cases of uncontrolled infection. Surgery may be indicated in cases of persistent vegetations, complications, or failure of medical management. The decision to repair or replace the pulmonary valve depends on the extent of damage and overall clinical condition [12,14,15]. While existing literature on long-term outcomes is limited, early diagnosis and appropriate management significantly impact prognosis. Follow-up studies assessing the cardiovascular health of TOF infants with a history of pulmonary valve vegetation are warranted.

## Conclusions

Pulmonary valve endocarditis occurring in isolation is infrequently observed in individuals with TOF. Nonetheless, it is crucial to uphold a degree of suspicion, particularly in cases with underlying risk factors, to ensure the timely identification of this rare condition. The significance of early diagnosis and intervention cannot be overstated as it contributes to improved prognoses in affected cases. Early diagnosis, vigilant monitoring, and tailored interventions, whether medical or surgical, play a pivotal role in improving outcomes for these vulnerable infants.
